# Supplementary material for: Chromothripsis during telomere crisis is independent of NHEJ, and consistent with a replicative origin
Source: Genome Res. 2019 May;29(5):737–49. doi: 10.1101/gr.240705.118 (PMC6499312; doi:10.1101/gr.240705.118)
Supplement: Supplemental Material [file supp_gr.240705.118_Supplemental_file_1.zip › contigs/annotated_contigs/DB46/contig.2.DB46_length_619_mean_cov_9.99192245557.docx]

**DB46_length_619_mean_cov_9.99192245557**

```
AGAAGGATTATAGGGTGGAGGAGCAGAGGCCAAGGAAGAATCGGAGCCTGATTTAGCTTGGCGGGGAGCGACCTGAAGAGGAGCAGTCT
 >chr20:1712730-1713057 + E=5e-182
GGGGAGGAGGGGAGAGGTCACATGGGTCAGTAGGAAAGGAAGATTGAAAAGACTCAGCGATGCTTGCGGTTGGGACTGAGGGAACAGGT

GGGGGGGAAAGAAGGAGGATTTGGGGTGAGTCACATTGGGAACAGAGACTAGGGAGGGACCGATGTGTAAAAGAATGCCTGGATGTCAG

GCACCTCAGACCATTTGCCCATTTTATGACAAGAATTATCTAGATCTTGTAGGATGGAGA|TCCTA|CATAATTCTTCATGAAAACACA
                                                                  >chr20:1710401-171068
CATGCTCTCCCTGCTGAGCATGTCCAGCTAAACTTTCAAACCCCAACCCCTTCTACAAAACAACAACTCCTTTCCTTCCTGGGCATGGT
8 - E=5e-161
TGGATAGTTTCACCTTTGGATACCTAGTTTTACCATCCTGACTAAACCATTATACAAACTCACAAAAACAAACCTAGCTGACCCCATAG

ATCCTAAATCCTTTCACCACTCATCTTTCCGTTCCTTAAAAACAGCCCTAGAAGCTGCTCTCACACTAGCTCTCCTTAACTCATCCC
```
